# Proficiency Testing of Viral Marker Screening in African Blood Centers — Seven African Countries, 2017

**DOI:** 10.15585/mmwr.mm6842a3

**Published:** 2019-10-25

**Authors:** Bakary Drammeh, Syria Laperche, Joan F. Hilton, Zhanna Kaidarova, Larisa Ozeryansky, Anindya De, Mireille Kalou, Irene Benech, Bharat Parekh, Edward L. Murphy, CDC Proficiency Testing Study Group, CDC Proficiency Testing Study Group, Zaituni Abdallah, Abby Abdikadir, Usman Ali Medugu Abjah, Oluwafemi Adegbamigbe, Victoria Adeleke, Lara Adeyeye, Janet Agba, Stephen Ajala, Sheila Allotey, Peter Paul Bacheyie, Patrick Banda, Obasi Barnabas, Oriji O. Bassey, Diocleciano Bila, Frank Bonsu, Remi Caparros, Aglean Chelimo, Charles Chilambula, Lameck Chinkango, Armando Chongo, Francelino Luciano Chongola, Onyeka Paul Chuka, Samuel Cobbson, Leonardo Desousa, Elliot Eli Dogbe, Augusto Domingos, Vanetine Ebomwonyi, Rumji Elisha, Joanisse Samuel Escova, Esperança Fideliz, Jerry Gwamna, Dunstan Haule, Daniel Hindes, Tehreen Ismail, Rui Labissone Jemusse, Alberto João, Muluken Kaba, Nasibu Kabolile, Zachary Kibet, Sammy Kihara, Basilius Kilowoko, Daniel Kimani, Steve Kimanzi, Martha Kimamo, Richard Kinyaha, Nick Kiptanui, Khamisi Kithi, Festus Koech, Steve Kunyenga, Yusto Kyando, Alexander Lawrence, Chimwemwe Limited, Jorge Lucio, Simon Manu, Sylvester Mattunda, Nassim Mbarak,, Bridon Mbaya, Alice Mbui, Japheth Mdenyo, Rodgers MC Mengwa, Chidozie Meribe, Thom Mfune, Michael Anthony Onoja, Fernando José Muria, Andrew Mwamtobe, Musa Mwamzuka, Christina Mwangi, Deeps Mwenebanda, Allan Mungai, Antony Mungai, Jabir Muhsin, Venantia Mwajombe, Charles Mwiyuma, Emmanuel Nani, Henry Ndaki, Olivier Ndahiriwe, Daniel Ndhlovu, Macrina Nditi, Miguel Neves, Eviness Ngwira, Bernard Nkrumah, Peter Nzioka, Kingsley Odiabara, Elizabeth Odthiambo, Omo T. Ojo, McPaul Okoye, Mavis Okyere, Ogunkola Oluyemisi, Anthony Owusu-Ansah, John Provinseh, Thomas Rotich, Razak Saasi, Simon Sabaya, Tinache Gabriel Sabonete, Yaw Sam, Ibrahim Sani, Bamidele Sunday, Priscilla Tarimo, Adekoya Benson Tolulope, Peter Torokaa, Ndeonasia Towo, Erlinda Umoru, Jose Victorino, Kingsley Wuor, James Yelima, Samuila Yohanna

**Affiliations:** ^1^Division of Global HIV and TB, Center for Global Health, CDC; ^2^Département des Agents Transmissibles par le Sang, Centre National de Référence Risque Infectieux Transfusionnels, Institut National de la Transfusion Sanguine, Paris, France; ^3^Department of Epidemiology & Biostatistics, University of California, San Francisco; ^4^Vitalant Research Institute, San Francisco, California, ^5^Department of Laboratory Medicine, University of California, San Francisco.; Western Zone Blood Transfusion Center Lab, Tanzania; Wajir District, Kenya; University of Maiduguri Teaching Hospital, Nigeria; Blood Transfusion Service, Federal Teaching Hospital, Ido-Ekiti, Nigeria; National Blood Transfusion Service Ibadan Centre, Nigeria; Federal Medical Centre Abeokuta, Nigeria; National Blood Transfusion Service Abuja Centre, Nigeria; National Blood Transfusion Service Kaduna Centre, Nigeria; Southern Accra Area Blood Center, Ghana; Tamale, Ghana; Katete Community Hospital, Malawi; National Blood Transfusion Service Owerri Centre, Nigeria; CDC, Nigeria; Hospital Rural de Chokwe, Mozambique; Kumasi South Hospital, Ghana; Institut National de la Transfusion Sanguine, Paris, France; Kenyatta National Hospital, Kenya; St. Peters Community Hospital, Likoma, Malawi; Mlambe Hospital, Malawi; Hospital Provincial de Manica, Mozambique; Hospital Provincial de Inhambane, Mozambique; University of Abuja Teaching Hospital, Nigeria; Methodist Faith Healing Hospital, Ghana; CDC, Mozambique; Komfo Anokye Teaching Hospital; Central Area Blood Centre, Ghana; Hospital Central de Maputo, Mozambique; National Blood Transfusion Service Benin Centre, Nigeria; National Blood Transfusion Service Jos Centre, Nigeria; Hospital Central da Beira, Mozambique; Hospital Provincial de Tete, Mozambique; CDC, Nigeria; National Blood Transfusion Service, Tanzania; Vitalant Research Institute, San Francisco, California; Aga Khan Hospital, Tanzania; Hospital Distrital de Madimba, Mozambique; Hospital Provincial de Quelimane, Mozambique; CDC, Malawi; Tanzania People’s Defense Force Blood Transfusion Center Lab, Tanzania; St. Mary’s Langata, Kenya; Regional Blood Transfusion Centre Embu, Kenya; St. Benedict Ndanda Hospital, Tanzania; CDC, Kenya; Regional Blood Transfusion Centre Embu, Kenya; Mater Mission Hospital, Kenya; Kibong’oto Hospital, Tanzania; Regional Blood Transfusion Centre Nakuru, Kenya; Regional Blood Transfusion Centre Mombasa, Kenya; Regional Blood Transfusion Centre Eldoret, Kenya; Nsanje District Hospital, Malawi; Ikonda Mission Hospital, Tanzania; Federal Medical Centre Lokoja, Nigeria; Bwaila Hospital, Malawi; Servico Nacional de Sangue, Mozambique; Southern Accra Area Blood Center, Ghana; CDC, Tanzania; Sayyidah Fatima, Kenya; Malawi Blood Transfusion Service, Malawi; Kenya National Blood Transfusion Service, Kenya; Bomu Hospital, Kenya; Ekwendeni Hospital, Malawi; CDC, Nigeria; Malawi Blood Transfusion Service, Malawi; Benue State University Teaching Hospital Makurdi, Nigeria; Hospital Distrital de Nacala, Mozambique; Atupele Community Hospital, Malawi; Bomu Hospital, Kenya; CDC, Rwanda; David Gordon Memorial Hospital, Malawi; Coptic Mission, Kenya; Presbyterian Church of East Africa Kikuyu Mission, Kenya; Amana Hospital, Tanzania; Southern Highlands Zone Blood Transfusion Center Lab, Tanzania; Southern Zone Blood Transfusion Center Lab, Tanzania; Dangme East District Hospital, Ghana; Lake Zone Blood Transfusion Center Lab, Tanzania; National Center for Blood Transfusion, Rwanda; Malawi Blood Transfusion Service, Malawi; Mafinga District Hospital, Tanzania; Centro de Referência Nacional de Sangue, Mozambique; St. Montfort Hospital, Malawi; CDC, Ghana; Pandya Hospital, Kenya; National Blood Transfusion Service Headquarters, Nigeria; Regional Blood Transfusion Centre Kisumu, Kenya; Olabisi Onabanjo University Teaching Hospital Sagamu, Nigeria; CDC, Nigeria; National Blood Service, Ghana; National Blood Transfusion Service Abeokuta Centre, Nigeria; Mankranso Government Hospital, Ghana; Tepa Government Hospital, Ghana; Regional Blood Transfusion Centre, Eldoret, Kenya; Nkawie-Toase Government Hospital, Ghana; Arusha Lutheran Medical Center, Tanzania; Hospital Distrital de Chiure, Mozambique; Konongo-Odumasi Government Hospital, Ghana; Usman Dan Fodio University Teaching Hospital, Sokoto, Nigeria; National Blood Transfusion Service Ado-Ekiti Centre, Nigeria; Northern Zone Blood Transfusion Center Lab, Tanzania; Ekiti State University Teaching Hospital Ado-Ekiti, Nigeria; Dodoma Regional Hospital, Tanzania; Eastern Zone Blood Transfusion Center Lab, Tanzania; National Blood Transfusion Service Lokoja Centre, Nigeria; Servico Nacional de Sangue, Mozambique; Koforidua Government Hospital, Ghana; National Blood Transfusion Service Maiduguri Centre, Nigeria; National Blood Transfusion Service Jalingo Centre, Nigeria

A 2014 report evaluating accuracy of serologic testing for transfusion-transmissible viruses at African blood center laboratories found sensitivities of 92%, 87%, and 90% for detecting infections with human immunodeficiency virus (HIV), hepatitis B virus (HBV), and hepatitis C virus (HCV), respectively (*1*). Following substantial investments in national blood transfusion service (NBTS) laboratories, in 2017 investigators tested proficiency at 84 blood center laboratories (29 NBTS and 55 non-NBTS) in seven African countries. A blinded panel of 25 plasma samples was shipped to each participating laboratory for testing with their usual protocols based on rapid diagnostic tests (RDTs) (*2*) and third and fourth generation enzyme immunoassays (EIA-3 and EIA-4). Sensitivity and specificity were estimated using separate regression models that clustered assays by laboratory and adjusted for assay type and NBTS laboratory status. Mean specificities were ≥95% for all three viruses; however, mean sensitivities were 97% for HIV-positive, 76% for HBV-positive, and 80% for HCV-positive samples. Testing sensitivities for all viruses were high when EIA-3 assays were used (≥97%). Lower sensitivities for HBV-positive samples and HCV-positive samples were associated with assay types other than EIA-3, used primarily by non-NBTS laboratories. Proficiency for HIV testing has improved following international investments, but proficiency remains suboptimal for HBV and HCV testing. In sub-Saharan African blood centers, the quality of rapid tests used for HBV and HCV screening needs to be improved or their use discouraged in favor of EIA-3 tests.

This cross-sectional study of blood transfusion laboratories was conducted in Ghana, Kenya, Malawi, Mozambique, Nigeria, Rwanda, and Tanzania during February–September 2017. A stratified sampling strategy targeting all NBTS laboratories and 10 non-NBTS laboratories per country (except Rwanda which has no non-NBTS laboratories) was used. Within each country, all non-NBTS laboratories were sorted by number of blood units tested annually, and five laboratories were chosen randomly from strata above and below the median. Assay types in use at study laboratories were RDT; EIA-3, which detects antibody or antigen; and EIA-4, which detects both antigen and antibody. Characteristics of participating NBTS and non-NBTS laboratories were compared by country, prevalence of assay types, and measures of laboratory expertise, such as annual volume of specimens tested.

Panels of 25 challenge specimens were prepared and characterized by the Institut National de la Transfusion Sanguine (Paris, France). Each panel included seven negative controls; seven specimens that contained HIV antigen and anti-HIV antibody (six HIV-1 and one HIV-2) (HIV-positive samples); six specimens containing hepatitis B surface antigen (confirmed by neutralization assay and quantified) (HBV-positive samples); and five specimens that contained HCV RNA and anti-HCV antibody (HCV-positive samples). All positive challenge specimens included viral genotypes that were specific to Africa. Plasma specimens were diluted with uninfected plasma to obtain specific antigen or antibody concentrations. The panels were confirmed to match their labels (Supplementary Table, https://stacks.cdc.gov/view/cdc/82012) at the Institut National de la Transfusion Sanguine, coded to allow for blinded testing, and sent to national coordinators who distributed them to participating laboratories while maintaining the cold chain.

Laboratories tested each challenge specimen in the panel using three assays, each designed to detect infection with HIV, HBV, or HCV, and reported findings for each assay. The primary study outcome was classification of each assay finding as correct or incorrect relative to each specimen’s true infection status; classification was done at the unblinded data analysis center. Sensitivity (correct detection of infection-positive status whether by antibody, antigen, or RNA) was estimated using approximately 25% of specimens for which the challenge virus matched the assay virus (seven HIV, six HBV, and five HCV), and specificity (correct detection of infection-negative status) was estimated using approximately 75% of specimens for which the challenge virus (or control) did not match the assay virus (18 HIV, 19 HBV, and 20 HCV).

The investigators used separate generalized estimating equation logit-binomial models to estimate mean sensitivity and specificity and 95% confidence intervals (CIs), each as a function of the three assay viruses (HIV, HBV, and HCV), clustering outcomes within laboratories. Multivariable models added NBTS status, assay type (RDT, EIA-3, or EIA-4), and all two-way interaction terms to the unadjusted model. The unadjusted model of specificity also included the identity of the challenge virus. All analyses were performed using SAS software (version 9.4; SAS Institute).

## Proficiency Testing

**Laboratory characteristics**. Among the seven countries, the number of participating laboratories ranged from one (Rwanda) to 20 (Nigeria), and the proportion that were NBTS laboratories ranged from 9% (Malawi and Mozambique) to 100% (Rwanda) (Table 1). Five non-NBTS laboratories (two each in Tanzania and Ghana and one in Kenya) did not participate, citing lack of reagents as the reason. Of 84 participating laboratories, 70 provided 100% of findings (25 specimens × three assays per laboratory), eight provided 93%, and six (all non-NBTS) provided 46%.

**TABLE 1:** Characteristics of participating blood centers and their laboratories, by National Blood Transfusion Service (NBTS) status — seven African countries, 2017

Characteristic	No. (%)	
	Non-NBTS laboratories* (N = 55)	NBTS laboratories (N = 29)
**Country**		
Ghana	8 (73)	3 (27)
Kenya	9 (60)	6 (40)
Malawi	10 (91)	1 (9)
Mozambique	10 (91)	1 (9)
Nigeria	10 (50)	10 (50)
Rwanda	0 (0)	1 (100)
Tanzania	8 (53)	7 (47)
**Type of HIV assay evaluated**		
Rapid diagnostic test	45 (82)	3 (10)
EIA-3	2 (4)	4 (14)
EIA-4	8 (15)	22 (76)
**Type of HBV assay evaluated^†^**		
Rapid diagnostic test	44 (80)	3 (10)
EIA-3	8 (15)	26 (90)
Unknown	3 (5)	0 (0)
**Type of HCV assay evaluated^†^**		
Rapid diagnostic test	43 (78)	3 (10)
EIA-3	6 (11)	17 (59)
EIA-4	1 (2)	9 (31)
Unknown	5 (9)	0 (0)
**Blood units assayed per year, median (25th, 75th percentiles)**	1,100 (192, 2,657)	11,000 (3,303, 22,800)
**Blood units produced per year**		
0	36 (65)	10 (34)
80–4,999	11 (20)	7 (24)
5,000–78,800	7 (13)	12 (41)
**Percentage of collections from volunteer donors, median (25th, 75th percentiles)**	10 (5, 60)	85 (75, 100)
**No. of laboratory personnel, median (25th, 75th percentiles)**	8 (5, 14)	4 (4, 7)
**Director has MD or PhD**	12 (22)	7 (24)
**Participates in EQAS program**	41 (75)	26 (90)

**Abbreviations:** EIA-3 = third generation enzyme immunoassay; EIA-4 = fourth generation enzyme immunoassay; EQAS = external quality assurance services; HBV = hepatitis B virus; HCV = hepatitis C virus; HIV = human immunodeficiency virus.

* Rwanda had no non-NBTS laboratories. Other participating countries had 10 each; in total, five failed to provide results, citing lack of reagents.

^†^ Sensitivity evaluations for assay targets HIV, HBV, and HCV were based on 84, 81, and 79 laboratories, respectively, because no assay was reported for HBV-positive specimens (three laboratories) and HCV-positive specimens (five laboratories).

Among NBTS laboratories, 90% used EIA-3 or EIA-4 assays, whereas among non-NBTS laboratories, 78%–82% used RDT assays. NBTS centers tested approximately 10 times more blood units than did non-NBTS laboratories, and higher proportions of NBTS than non-NBTS laboratories produced blood components (66% versus 35%) and received blood primarily from volunteer donors (100% versus 60%).

**Sensitivity.** Unadjusted mean sensitivity for detecting HIV-positivity was 97% (95% CI = 95%–98%); for detecting HBV-positivity was 76% (95% CI = 71%–81%); and for detecting HCV-positivity was 80% (95% CI = 75%–86%) (Table 2). Sensitivity exceeded 90% for HIV-positive detection in all seven countries; however, this level of sensitivity for identifying HBV-positive specimens was reached only in Kenya and Rwanda, and for HCV-positive specimens, only in Kenya, Mozambique, and Rwanda (p<0.001). At NBTS laboratories, all three assays’ sensitivities to their respective target viruses exceeded 92%; however, at non-NBTS laboratories, sensitivity to HBV-positive was 66% and to HCV-positive was 74% (p<0.001). Statistically significantly higher levels of testing sensitivity were observed in laboratories that tested more blood donations per year (p = 0.006), produced more components per year (p = 0.026), and had higher percentages of donors who were volunteers (p = 0.013). Testing sensitivity was not associated with the number of laboratory personnel.

**TABLE 2:** Sensitivity* for detecting evidence of infection with human immunodeficiency virus (HIV), hepatitis B virus (HBV), and hepatitis C virus (HCV), by selected characteristics of 84 laboratories — seven African countries, 2017

Characteristic	Assay target virus (no. of laboratories^†^) Mean % (95% CI)			p-value^§^
	HIV (n = 84)	HBV (n = 81)	HCV (n = 79)	
**Overall, unadjusted**	**96.6 (95.0–98.1)**	**75.8 (70.8–81.2)**	**80.2 (74.7–86.2)**	**—**
**Country** ^¶^				
Ghana	93.5 (87.8–96.6)	58.5 (52.9–63.8)	70.9 (50.8–85.2)	<0.001
Kenya	99.0 (93.8–99.9)	93.3 (84.2–97.4)	96.0 (89.3–98.6)	
Malawi	98.7 (92.0–99.8)	60.6 (47.4–72.4)	60.0 (43.2–74.7)	
Mozambique	98.7 (91.9–99.8)	54.7 (42.1–66.8)	94.0 (85.1–97.7)	
Nigeria	98.5 (94.7–99.6)	82.5 (69.6–90.7)	78.8 (61.6–89.6)	
Rwanda	100	100	100	
Tanzania	90.5 (83.1–94.8)	84.3 (69.6–92.7)	75.4 (63.4–84.4)	
**Assay type**				
Rapid	95.0 (91.9–96.9)	59.8 (54.7–64.6)	70.5 (61.1–78.4)	<0.001
EIA-3	97.7 (84.7–99.7)	98.0 (91.4–99.6)	96.9 (92.2–98.8)	
EIA-4	99.0 (96.8–99.7)	(Not used)	84.4 (74.3–91.0)	
**NBTS**				
No	95.5 (92.8–97.3)	66.2 (60.2–71.7)	73.8 (64.7–81.2)	<0.001
Yes	98.5 (95.8–99.5)	93.0 (83.4–97.3)	91.8 (86.7–95.0)	
Blood units tested per year**				
1,000	96.6 (94.7–97.8)	75.3 (69.8–80.2)	79.5 (72.9–84.8)	0.006
3,162	97.1 (95.3–98.3)	79.3 (73.6–84.1)	82.0 (75.6–87.1)	
10,000	97.6 (95.7–98.7)	82.8 (76.6–87.6)	84.3 (77.5–89.4)	
Components produced per year**				
None	95.5 (92.4–97.4)	73.3 (65.9–79.5)	74.4 (65.1–82.0)	0.026
1,000 blood units	97.6 (95.4–98.7)	78.8 (71.3–84.4)	85.2 (78.3–90.1)	
10,000 blood units	98.0 (95.4–99.2)	80.5 (70.7–87.6)	87.8 (79.9–92.9)	
**Percentage of donors who are volunteers**				
1–24	96.2 (92.8–98.0)	69.3 (61.7–75.9)	69.8 (57.8–79.6)	0.013
25–74	94.4 (88.9–97.3)	64.2 (51.4–75.2)	89.2 (79.3–94.6)	
75–100	98.2 (94.3–99.4)	89.8 (81.0–94.8)	85.3 (76.0–91.4)	
**No. of laboratory personnel**				
1–6	97.6 (95.4–98.8)	74.7 (66.0–81.8)	81.7 (73.2–87.9)	0.36
7–54	95.3 (91.7–97.4)	77.9 (70.7–83.7)	78.1 (67.7–85.9)	

**Abbreviations:** CI = confidence interval; EIA-3 = third generation enzyme immunoassay; EIA-4 = fourth generation enzyme immunoassay; NBTS = national blood transfusion service.

* Based on univariate models.

^†^ Because HBV- and HCV-positive specimens were not assayed by three and five laboratories, respectively, sensitivity evaluations for assay targets HIV, HBV, and HCV were based on 84, 81, and 79 laboratories, respectively.

^§^ P-values report statistical significance of associations of sensitivity with the interaction between assay virus and laboratory characteristics.

^¶^ Model excluded Rwanda and excluded the interaction term. P-value reports statistical significance of association of sensitivity with country.

** Characteristic was analyzed on the log-10 scale. Mean sensitivity was estimated at the values shown.

Based on the multivariable model, adjusted sensitivities uniformly exceeded 96% when EIA-3 was used; however, the sensitivity of EIA-4 to detect HCV-positivity was <85%, and RDT assay sensitivities to detect HBV- and HCV-positivity were <71%. Sensitivity for detecting HIV-positivity was ≥95% regardless of laboratory or assay type. Sensitivity varied significantly among assay types (p = 0.011) but not among assay target viruses (p = 0.30) or between NBTS laboratory status (p = 0.81), and none of the three pairwise interaction effects was statistically significant (p≥0.25). These findings are reflected by observed sensitivity proportions (Figure) that show that EIA-3 assays performed equally well or better than others for detecting HIV-, HBV-, and HCV-positivity, regardless of NBTS status.

**FIGURE:**
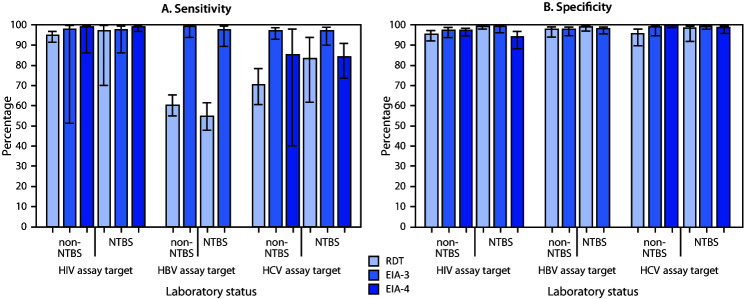
Adjusted mean estimates of sensitivity (A) and specificity (B) for identification of positive and negative challenge specimens for human immunodeficiency virus (HIV), hepatitis B virus (HBV), and hepatitis C virus (HCV), by assay virus, assay type, and National Blood Transfusion Services (NTBS) laboratory status — seven African countries,* 2017† **Abbreviations: **EIA-3 = third generation enzyme immunoassay; EIA-4 = fourth generation enzyme immunoassay; RDT = rapid diagnostic test. * Ghana, Kenya, Malawi, Mozambique, Nigeria, Rwanda, and Tanzania. ^^† ^^95% confidence intervals indicated by error bars.

**Specificity**. Unadjusted mean testing specificity was 95% (95% CI = 93%–97%) for HIV-negative specimens, 96% (95% CI = 93%–98%) for HBV-negative specimens, and 95% (90%–98%) for HCV-negative specimens. Across all assay target viruses, mean specificity was 90%–92% in three countries (Malawi, Mozambique, and Tanzania) and ≥98% in the other four countries.

Adjusted estimates based on the multivariable model showed that the targeted assays varied in specificity by assay type (p = 0.054) and interaction with NBTS status (p = 0.058). Specificity was relatively low at non-NBTS laboratories for RDT assays targeting HCV or HIV and at NBTS laboratories for EIA-4 assays targeting HIV (Figure).

## Discussion

This investigation of testing proficiency of targeted assays for HIV, HBV, and HCV found specificities to be high overall, with clinically negligible variations by NBTS status or assay type. In contrast, clinically important variation in sensitivities within and between assay targets was found. The finding that non-EIA-3 tests had lower sensitivity than did other assay types for detecting HBV- and HCV-positive specimens but not HIV-positive specimens is consistent with findings from previous studies (*1*–*4*). As noted, variation in testing proficiency for sensitivity among countries primarily reflects variation among assay types rather than between NBTS and non-NBTS laboratories.

This study found higher sensitivity for detecting HIV-positivity but lower sensitivity for detecting HBV- and HCV-positivity than is generally associated with the use of RDTs, compared with previous studies using similar methods (*1*,*2*). These results suggest that RDT assays targeting HIV perform better or have better quality assurance than do RDT assays targeting the hepatitis viruses. The poorer performance of RDT assays for detecting HBV- and HCV-positivity is most likely attributable to the quality of the assays themselves, because deficiency in performing the tests could have been signaled by lower mean accuracy at non-NBTS compared with NBTS laboratories. Of note, lower sensitivity to HCV-positivity using the EIA-4 was limited to a single reputable assay, suggesting a need to rule out poor technical performance or recording errors. After all laboratories had completed testing and the CDC International Laboratory Branch had evaluated the results, it conducted site visits at low-performing laboratories and developed recommendations for remediation.

The findings in this report are subject to at least four limitations. First, the numbers of positive-challenge specimens per assay target virus were small, which resulted in few response levels for sensitivity estimations. Second, the positive samples were diluted to approximate difficult samples, but this limits extrapolation of operational sensitivity. Third, the investigators attempted to overcome sampling bias by using a random sample of non-NBTS laboratories; however, five of these laboratories failed to participate in the study, and six others submitted incomplete data, which suggests problems with their supplies of assay kits. Finally, the unanticipated strong association of assay type with NBTS status and few NBTS laboratories per country precluded fully distinguishing the effects of assay type, NBTS status, and country.

Variation in blood center laboratory proficiency among sub-Saharan African countries has been reported previously and likely relates to both assay quality, representing a range of manufacturers, and organizational structures, resources, and training of technicians (*5*–*7*). Future studies of testing proficiency could be designed to study manufacturers in addition to assay type, with the aim of identifying products that perform poorly. Alternatively, future study protocols could provide high-accuracy assay kits targeting HIV, HBV, and HCV to better distinguish between assay quality and operator error.

To ensure that transfusion-transmitted viruses in donated blood are detected, the use of rapid diagnostic tests for HBV and HCV should be discouraged because of the general suboptimal performance of these assays. Where possible, scarce blood center resources should be allocated to enable all blood center laboratories to use EIA-based assays from selected manufacturers, improve the reliability of supply chains and implement standard quality assurance protocols for conducting the assays, and require technical staff members to participate in testing-proficiency training programs. However, quality improvements might be difficult to sustain if African national budgets are not supplemented by international funding (*8*).

**Summary**

**What is already known about this topic?**

Substantial international investments have been made in African national blood transfusion services (NBTS) following reports of deficiencies in viral marker screening at African blood center laboratories.

**What is added by this report?**

Standardized proficiency testing conducted in seven African countries during 2017 found that proficiency in human immunodeficiency virus testing has improved, but testing proficiency for hepatitis B virus (HBV) and hepatitis C virus (HCV) needs to be improved.

**What are the implications for public health practice?**

Most poor performance in hepatitis virus testing can be attributed to the use of rapid tests rather than the non-NBTS setting of the laboratories. Remediation should be focused on improving the quality of rapid tests or avoiding their use.

## References

[1] Bloch EM, Shah A, Kaidarova Z, ; Anglophone Africa Transfusion Research Group. A pilot external quality assurance study of transfusion screening for HIV, HCV and HBsAG in 12 African countries. Vox Sang 2014;107:333–42. 10.1111/vox.12182 25052195 PMC4205185

[2] Pruett CR, Vermeulen M, Zacharias P, Ingram C, Tayou Tagny C, Bloch EM. The use of rapid diagnostic tests for transfusion infectious screening in Africa: a literature review. Transfus Med Rev 2015;29:35–44. 10.1016/j.tmrv.2014.09.003 25447555

[3] Laperche S; Francophone African Group for Research in Blood Transfusion. Multinational assessment of blood-borne virus testing and transfusion safety on the African continent. Transfusion 2013;53:816–26. 10.1111/j.1537-2995.2012.03797.x 22804482

[4] Prugger C, Laperche S, Murphy EL, Screening for transfusion transmissible infections using rapid diagnostic tests in Africa: a potential hazard to blood safety? Vox Sang 2016;110:196–8. 10.1111/vox.12327 26646317 PMC5061037

[5] Tagny CT, Diarra A, Yahaya R, Characteristics of blood donors and donated blood in sub-Saharan Francophone Africa. Transfusion 2009;49:1592–9. 10.1111/j.1537-2995.2009.02137.x 19389036

[6] Tagny CT, Kouao MD, Touré H, Transfusion safety in francophone African countries: an analysis of strategies for the medical selection of blood donors. Transfusion 2012;52:134–43. 10.1111/j.1537-2995.2011.03391.x 22014098 PMC3668689

[7] Bloch EM, Vermeulen M, Murphy E. Blood transfusion safety in Africa: a literature review of infectious disease and organizational challenges. Transfus Med Rev 2012;26:164–80. 10.1016/j.tmrv.2011.07.006 21872426 PMC3668661

[8] Adepoju P. Blood transfusion in Kenya faces an uncertain future. Lancet 2019;394:997–8. 10.1016/S0140-6736(19)32140-3 31544753

